# Noninvasive studies may have potential to replace cystoscopy in non-muscle invasive bladder cancer follow-up

**DOI:** 10.1038/s41598-022-23111-1

**Published:** 2022-12-15

**Authors:** Jongsoo Lee, Ji Eun Heo, Sung Ku Kang, Kwang Suk Lee, Hyunho Han, Won Sik Jang, Young Deuk Choi

**Affiliations:** 1 Department of Urology, Urological Science Institute, Yonsei University College of Medicine, 50-1, Yonsei-Ro, Seodaemun-Gu, Seoul, 03722 Korea; 2 Department of Urology, National Health Insurance Service Ilsan hospital, Goyang, 10444 Korea

**Keywords:** Bladder cancer, Cancer imaging

## Abstract

Bladder cancer has a high recurrence rate which requires frequent follow-up. Cystoscopy is currently the gold standard for follow-up which is invasive and undesirable procedure for patients. We aimed to investigate the feasibility of noninvasive studies for follow-up of non-muscle invasive bladder cancer. This retrospective study was done for non-muscle invasive bladder cancer patients with abnormal lesion at follow up cystoscopy, therefore those needed transurethral resection of bladder tumor (TUR-BT). Inclusion criteria was patients who had preoperative bladder magnetic resonance imaging (MRI) within 1 month to TUR-BT and urine cytology results. MRI, urine cytology, and surgical pathology results were analyzed for sensitivity, specificity, positive and negative predictive values, accuracy, diagnostic odds ratio, and number needed to misdiagnose for the diagnostic performance of non-invasive studies. From total of 2,258 TUR-BT cases, 1,532 cases of primary TUR-BT and 481 cases which bladder MRI were not done was excluded. Finally, 245 cases of TUR-BT were included. Combined urine cytology and bladder MRI showed 96% sensitivity, 43% specificity, 89% positive and 67% negative predictive values, 87% accuracy, 16.2 diagnostic odds ratio, and 7.4 number needed to misdiagnose values. Among nine false-negative cases, three (1.2%) were missed by the radiologist, two (0.8%) had an empty bladder during magnetic resonance imaging, and three (1.2%) had gross hematuria which needed cystoscopy despite of bladder MRI or urine cytology result. Only one case (0.4%) was missed based on symptoms and noninvasive tests. However, none of the false-negative cases showed rapid extensive progression requiring radical or partial cystectomy. The combination of bladder MRI and urine cytology was comparable to cystoscopy for the follow-up of recurred lesions in non-muscle invasive bladder cancer patients for sensitivity, but not for specificity. However, it may reduce the need for cystoscopy and allowing patients to have choices for follow up diagnostic methods. Also, additional imaging tests to evaluate kidney, ureter and peri-vesical lesions can be reduced.

## Introduction

About 75% of patients with bladder cancer are initially diagnosed with non-muscle-invasive bladder cancer (NMIBC), of which 70–75% are stage Ta and 20–25% are stage T1 and about 5–10% are flat high-grade lesions^1^. Additionally, the recurrence of bladder cancer is very common. Routine follow-up cystoscopy is the gold standard test following transurethral resection of bladder tumors (TUR-BT). Although the recent dissemination of flexible cystoscopy has reduced patient discomfort during the test, it remains as an uncomfortable procedure performed while the patient is awake^2,3^. Moreover, antibiotics has to be administered for patients who has host related factors of infection according to the guideline^4^. To date, a test capable of replacing cystoscopy has not been established^5^. Even with the development of molecular diagnosis for urine-based early screening, diagnosis, and follow-up for bladder cancer, these tests cannot determine the range and depth of the cancer^6^.

Multi-parametric magnetic resonance imaging (mpMRI) improved diagnostic and staging accuracy of the imaging study and can reveal organs adjacent to the target organ. It is currently widely used, and its image quality is continuously being improved. In urinary system imaging, following the active use of Prostate Imaging-Reporting and Data System (PI-RADS), the recent development of Vesical Imaging-Reporting and Data System (VI-RADS) has led to the systemic reporting of bladder MRI^7^.

A large systematic review and meta-analysis study revealed that the sensitivity and specificity for the prediction of bladder cancer were 0.87–0.92 and 0.79–0.87, respectively^8–10^. In addition, in a recent study, the diagnostic performance of VI-RADS has been reported to have a sensitivity of 0.83 and specificity of 0.90^11^. In this study, we aimed to evaluate the ability of noninvasive test (urine cytology and mpMRI) to substitute cystoscopy for NMIBC follow-up and pathologically confirm the mpMRI findings.

## Methods

### Study population

**Figure 1:**
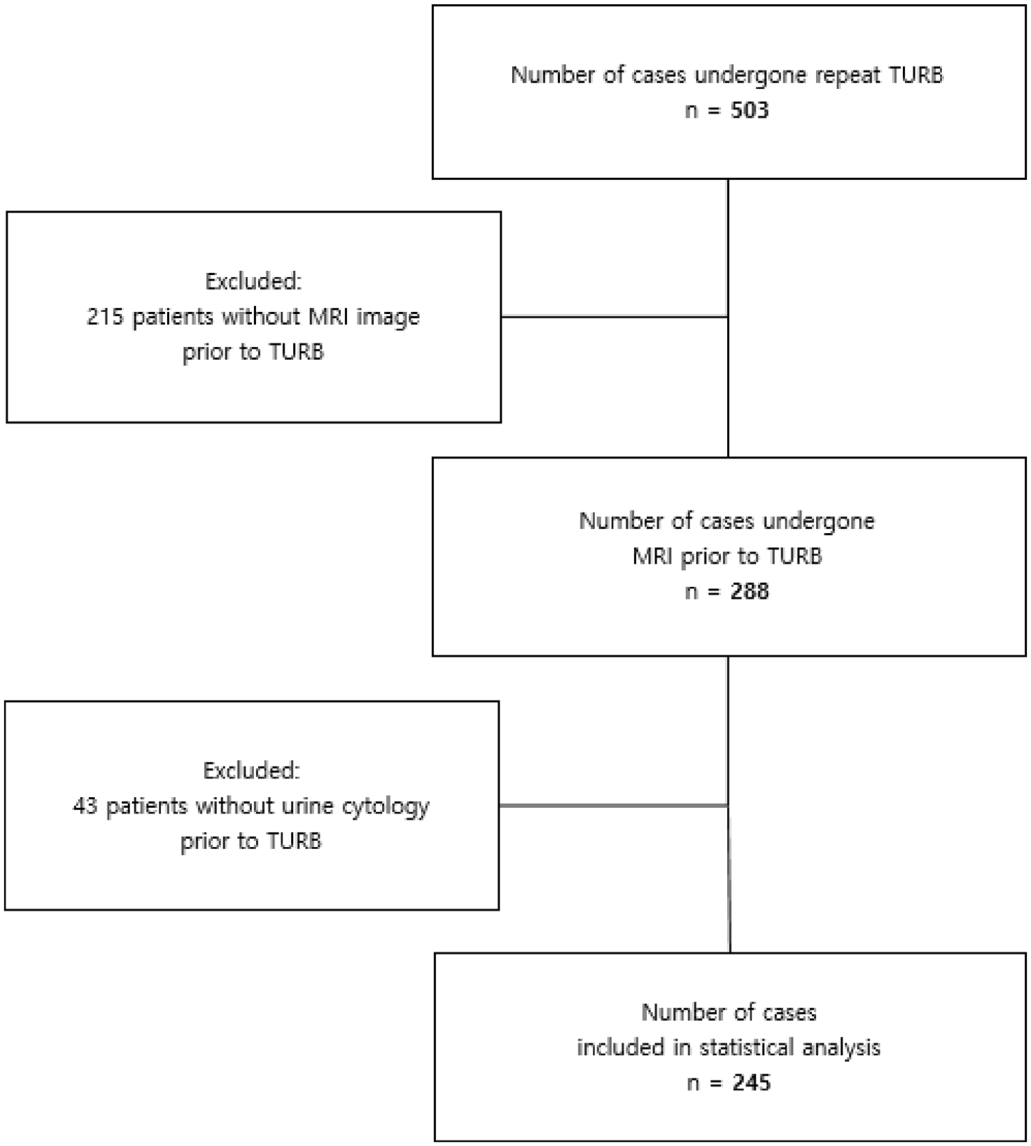
Flowchart of study population.

This retrospective study included cases of TUR-BT performed by a single surgeon (Y.D.C.) between May 2010 and May 2018. All decisions to perform TUR-BT were made based on cystoscopy. Cases of primary TUR-BT and cases of TUR-BT without urine cytology or preoperative bladder MRI within 1 month were excluded. After applying the exclusion criteria, 245 cases were included (Fig. 1).

### Multiparametric MRI

**Figure 2:**
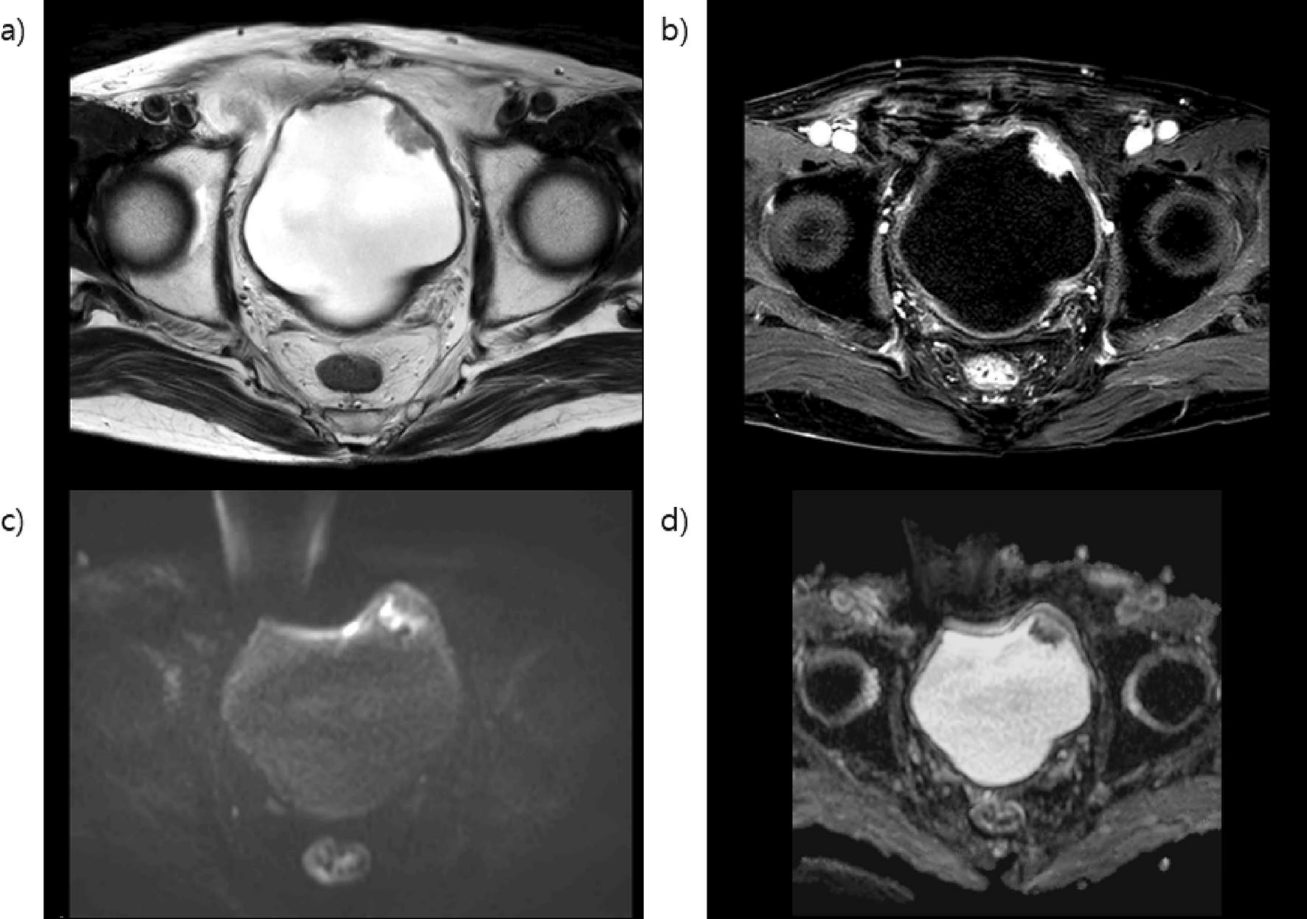
Magnetic resonance images of non-muscle-invasive bladder cancer (**a**) T2-weighted image in the left anterolateral aspect of the bladder wall showing a non-muscle-invasive papillary lesion preserving the hypointense detrusor lining underneath. (**b**) Dynamic contrast-enhanced imaging subtracted image displaying no early enhancement of muscularis propria. (**c**) Diffusion-weighted imaging (DWI) native image and (**d**) apparent diffusion coefficient (ADC) map showing tumor hyperintense on DWI and hypointense on ADC, with low SI thickened inner layer on DWI. The multiparametric magnetic resonance imaging patterns are consistent with non-muscle-invasive bladder cancer. *MRI* magnetic resonance imaging.

**Figure 3:**
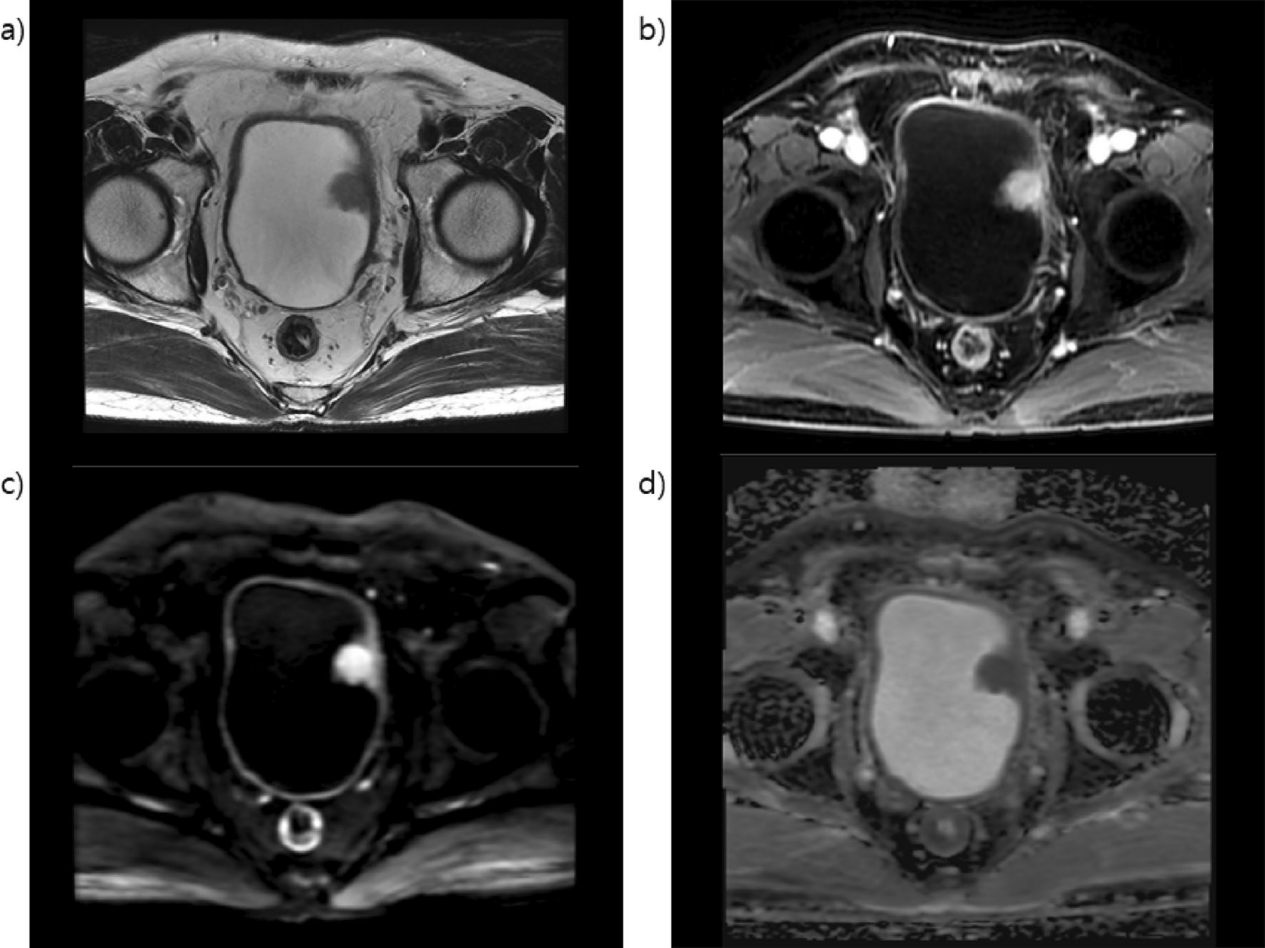
Magnetic resonance images of equivocal muscle invasive bladder cancer (**a**) T2-weighted image in the left lateral aspect of the bladder wall showing exophytic tumor without high SI thickened inner layer but with no clear disruption of low SI muscularis propria. (**b**) Early enhancement of muscularis propria but with no clear disruption of low SI muscularis propria. (**c**) Diffusion-weighted imaging native image and (**d**) apparent diffusion coefficient map showing muscularis propria with continuous intermediate SI but with no clear disruption of muscularis propria. The muscle layer invasion of tumor is equivocal.

**Figure 4:**
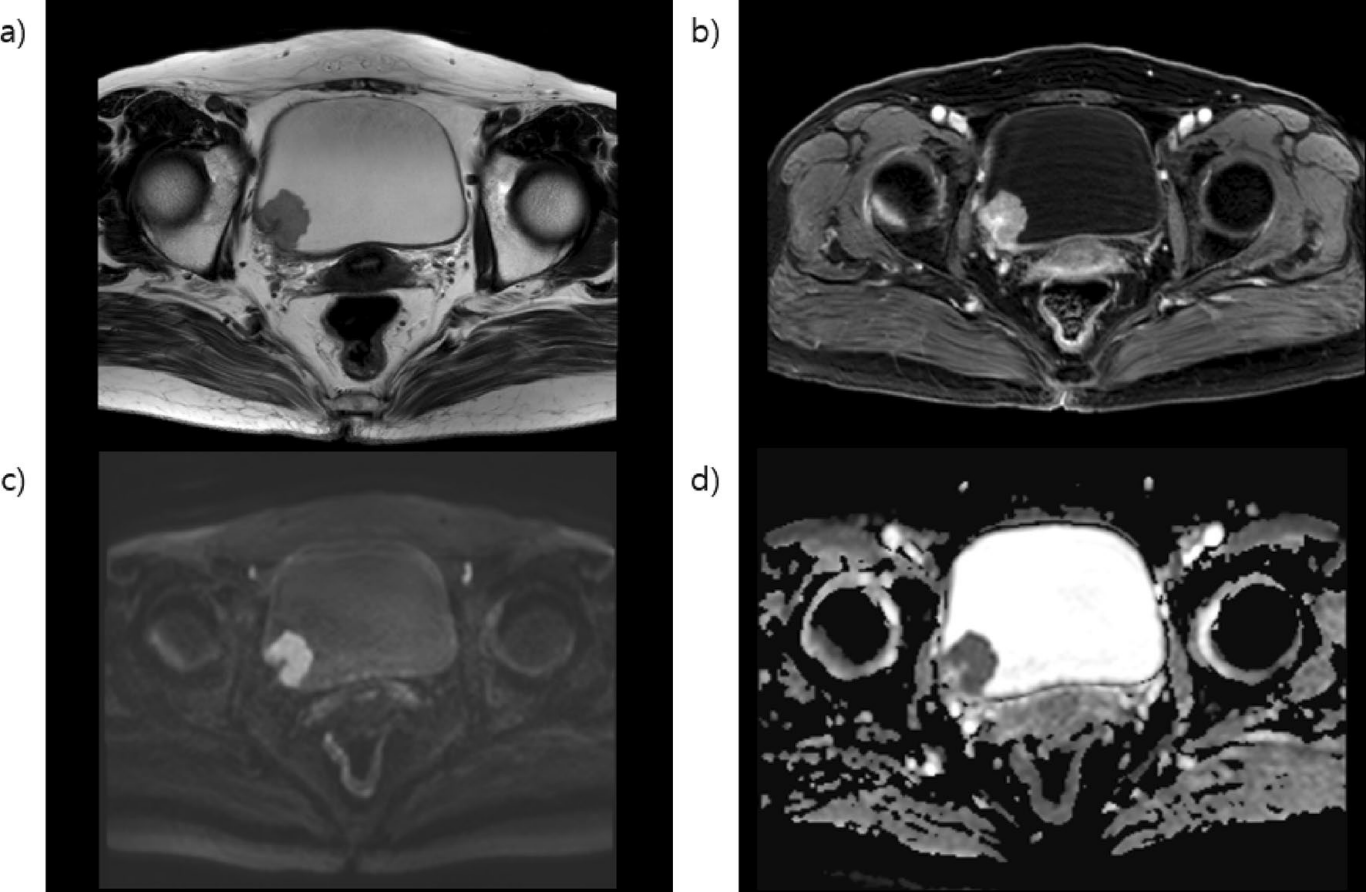
Magnetic resonance images of muscle-invasive bladder cancer (**a**) T2-weighted image in the right posterolateral aspect of the bladder wall showing tumor with stalk without high SI thickened inner layer with no clear disruption of low SI muscularis propria. (**b**) Early enhancement of muscularis propria with early enhancement of inner layer but with no clear disruption of low SI muscularis propria. (**c**) Diffusion-weighted imaging (DWI) native image and (**d**) apparent diffusion coefficient (ADC) map showing high SI tumor on DWI and low SI tumor on ADC extending focally to muscularis propria. The muscle layer invasion of the tumor is likely.

**Figure 5:**
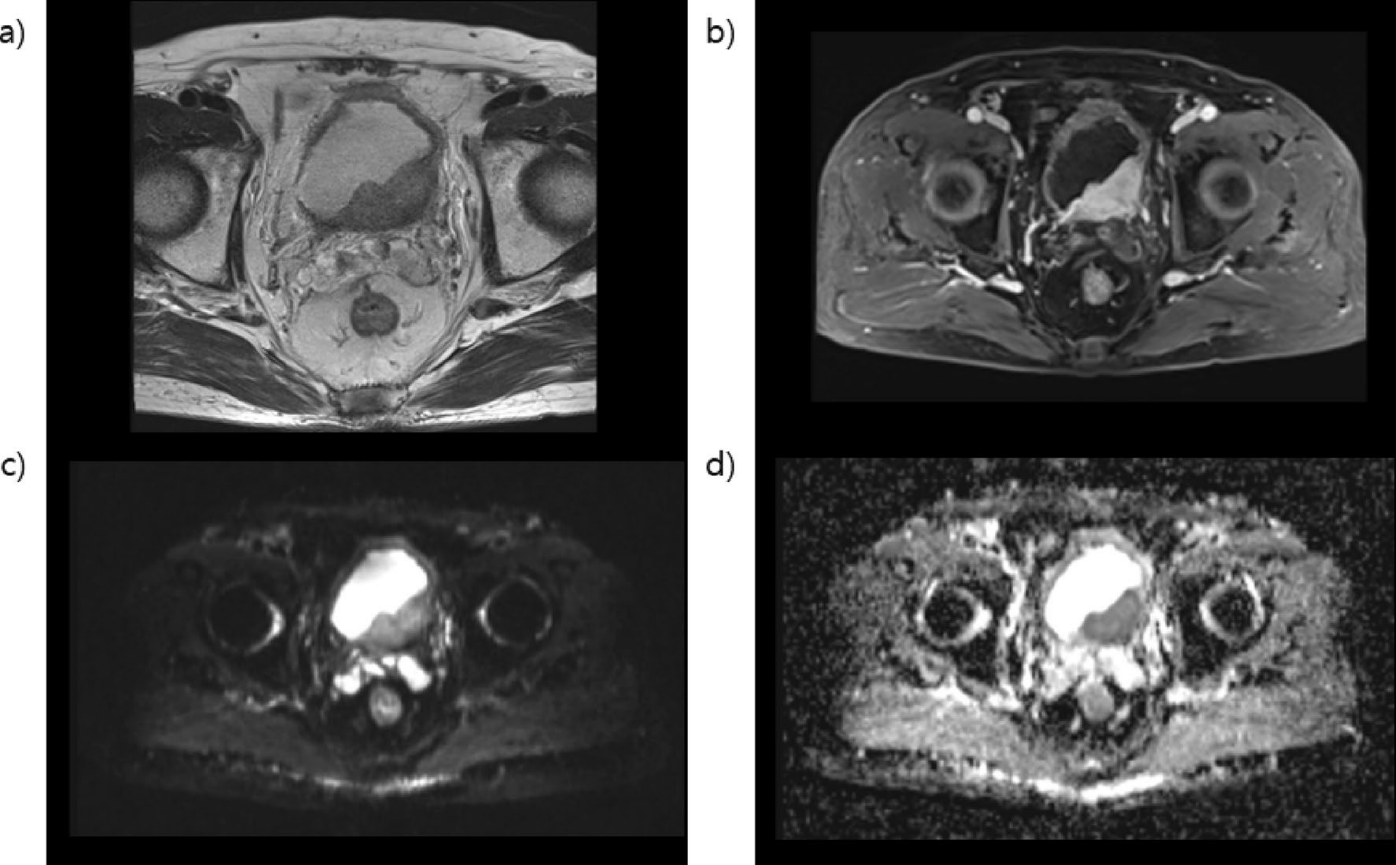
Magnetic resonance images of the extravesical tissue-invasive bladder cancer (**a**) T2-weighted image in the left posterolateral aspect of the bladder wall showing a bladder cancer invading underneath the hypointense detrusor lining to the extravesical fat tissue. (**b**) Dynamic contrast-enhanced image showing the enhancement of the lesion, invading the extravesical fat layer. (**c**) Diffusion-weighted imaging native image and (**d**) apparent diffusion coefficient map showing highly restricted diffusion of the mass with low signal intensity. There is an invasion of the entire bladder wall and extravesical fat. The multiparametric magnetic resonance imaging patterns are consistent with the extravesical tissue invasion of bladder cancer.

Multiparametric MRI images were obtained using 4-mm slice thickness for T1-weighted imaging, T2-weighted imaging, diffusion-weighted imaging (DWI), apparent diffusion coefficient (ADC) mapping, and dynamic contrast-enhanced imaging. Examination was performed with the use of a 1.5 T magnetic resonance scanner (Achieva, Philips Medical Systems, Best, The Netherlands) on four cases and a 3.0 T magnetic resonance scanner (Discovery MR750, GE Healthcare, Milwaukee, WI) for the rest of the cases. Interpretation of the mpMRI findings was performed by three genitourinary radiologists from the same institution. Patients were instructed not to void within approximately 2 hours to imaging, and patients with urinary catheters had their catheters clamped approximately 2 hours before the examination. The definition of staging for bladder tumor is explained in Figs. 2, 3, 4 and 5. Furthermore, interpretation of the MRI findings was performed by three professional genitourinary radiologists and was not repeated for this study. Interpretation of the MRI findings was performed preoperatively; hence, pathological reports did not affect the interpretation.

### Urine cytology, cystoscopy, and surgical pathology

Urine samples for cytology were collected prior to cystoscopy and were directly sent to the pathology department in the same institution. Interpretation of cytology was performed by professional pathologists with minimum of 7 years of experience, taking turns according to their duty. The results were reported regarding to The Paris System for reporting urine cytology, categorized as unsatisfactory, negative for high-grade urothelial carcinoma (HGUC), atypical urothelial cell, suspicious for HGUC, or HGUC. Follow-up cystoscopy was performed according to the National Comprehensive Cancer Network guideline for bladder cancer. General or spinal anesthesia was administered to patients undergoing TUR-BT. Bladder cancer staging was based on the 8th American Joint Committee on Cancer (AJCC) TNM staging system for both clinical and pathologic staging. The mentioned pTis lesion in our study was defined as pathologically confirmed Tis without synchronous pT1–4 lesions.

### Data collection

The patients’ clinical and pathological characteristic data were obtained from the institutional electronic medical record database. Data included patient age at surgery, number of prior TUR-BTs, clinical and pathological AJCC stage, urine cytology results, MRI results, clinical TNM stage, TUR-BT pathology results, type of intravesical therapy, and further definitive treatment.

### Statistical analysis

Bladder MRI results, urine cytology findings, and surgical pathology results were analyzed for sensitivity, specificity, positive and negative predictive values (PPV and NPV, respectively), accuracy, diagnostic odds ratio (DOR), and the number needed to misdiagnose (NNM). Clinicopathological data were summarized according to clinical T staging and urine cytology results. Subgroup analysis was performed using MRI, cytology, and combined for both. Fisher’s exact test and chi-squared test were used to compare categorical variables. Moreover, the Mann–Whitney *U*-test was used to compare continuous variables across dichotomous categories. Statistical analyses were performed using IBM SPSS software (version 23; IBM Corp., Armonk, NY). All tests were two-tailed, and a p value < 0.05 was considered statistically significant.

All research was performed in accordance with relevant guidelines/regulations. The requirement for written informed consent was waived (Yonsei University Health System Institutional Review Board/IRB No, 4-2020-0134) owing to the retrospective design of the study. This study was conducted in accordance with the Declaration of Helsinki and was approved by the Yonsei University Health System Institutional Review Board after a review of the study protocol (IRB No, 4-2020-0134).

## Results

### Baseline patient characteristics

We enrolled 245 TUR-BT cases who underwent preoperative urine cytology, MRI, and cystoscopy evaluations. All cases were diagnosed as NMIBC after previous TUR-BT. The median age at current surgery was 69 (Interquartile range (IQR), 62–75) years, and the patients underwent a median of 4 (IQR, 2–6) cystoscopies between the previous and current TUR-BT for NMIBC follow-up. Patient characteristics and clinical information are reported in Table 1.

**Table 1:** Descriptive statistics of baseline characteristics in patients with bladder cancer.

Sex (male, %)	217	89%
Age at initial diagnosis, years (IQR)	66	(58–73)
Age at current surgery, years (IQR)	69	(62–75)
Number of surgeries, n (IQR)	2	(2–3)
Number of cystoscopies before current surgery, n (IQR)	4	(2–6)
Interval from previous operation, months (IQR)	14	(7–29)
**Clinical staging, n (%)**		
T		
No lesion	52	21%
≤ T1	125	51%
≥ T2	68	28%
N1	9	4%
M1	4	2%
**Cytology, n (%)**		
Unsatisfactory	10	4%
Negative for HGUC	86	35%
Atypical urothelial cell	44	18%
Suspicious for HGUC	31	13%
HGUC	74	30%
**Pathologic T staging, n (%)**		
No tumor	42	17%
Tis	24	10%
≤ T1	134	55%
T2 ≤	45	18%
**Histologic grade, n (%)**		
Low	36	15%
High	143	58%
**Intravesical therapy before current TUR-BT, n (%)**		
None	55	22%
MMC	48	20%
Within 1 year ^a^	22	28%
BCG	142	58%
Within 1 year ^a^	59	41%
**Further definitive treatment, n (%)**		
Radical cystectomy	37	15%
Partial cystectomy	14	6%

*MMC* Mitomycin-C, *BCG* Bacillus Calmette-Guerin, *IQR* inter-quartile range;

^a^Interval between intravesical therapy and MRI, which has been dichotomized to less than 1 year and more than 1 year.

Preoperative bladder MRI revealed no abnormal lesions in 52 (21%) cases, whereas 125 (51%) cases had T1 lesions and 68 (28%) cases had T2 or higher-stage lesions. Urine cytology revealed unsatisfactory or negative for HGUC findings in 96 (39%) cases and atypical urothelial cells or worse findings in 149 (61%) cases.

The pathology reports of TUR-BT specimens revealed pathologic T staging lower than T1 and higher than T2 in 159 (54%) and 45 (18%) cases, respectively. Histologic analysis showed that 36 (15%) cases had low-grade and 145 (58%) cases had high-grade findings. Forty (16%) cases had carcinoma in situ (CIS), with 11 (4%) cases having only a CIS lesion without papillary or solid lesions.

### Categorizing clinical findings

Clinical staging according to bladder MRI and urine cytology results were organized according to pathologic T staging from TUR-BT specimens (Table 2). Among cases with a negative pathological result, 48% and 73% had no abnormal findings on MRI and bladder urine cytology, respectively. CIS lesions were associated with 54% of negative MRI findings and 21% of negative urine cytology findings. In pathologic Ta, T1 lesion group and T2 lesion group, abnormal findings in MRI were reported in 86% and 100%, while abnormal findings in cytology were reported in 67% and 73%, respectively.

**Table 2:** Pathologic T staging of TUR-BT specimens according to clinical diagnostic modalities.

	Overall (n = 245)		Pathologic T staging								
			T0 (n = 42)		Tis (n = 24)		Ta, T1 (n = 134)		≥ T2 (n = 45)		*P* value
	n	%	n	%	n	%	n	%	n	%	
**MRI T staging**											
No lesion	52	21	20	48	13	54	19	14	0	0	< 0.001
≤ T1	125	51	16	38	10	42	88	66	11	24	
≥ T2	68	28	6	14	1	4	27	20	34	76	
**Urine cytology**											
Unsatisfactory	10	4	1	2	0	0	6	4	3	7	< 0.001
Negative for HGUC	86	35	30	71	5	21	47	30	9	20	
Atypical urothelial cell	44	18	4	10	4	17	31	20	9	20	
Suspicious for HGUC	31	13	1	2	5	21	24	15	6	13	
HGUC	74	30	6	14	10	42	50	32	18	40	

*MRI* magnetic resonance imaging, *TUR-BT* transurethral resection of bladder tumor.

Clinical staging using bladder MRI and urine cytology was also organized according to histologic grading from TUR-BT specimens (Table 3). MRI findings with no abnormal lesions were noted in 14% of cases with low-grade lesions and 16% with high-grade lesions. The corresponding values for a negative urine cytology result were 81% and 22%, respectively. There was a significant association between preoperative MRI, urine cytology findings and pathologic, histologic staging based on Pearson’s chi-square test (Tables 2, 3, p < 0.001).

**Table 3:** Histologic grade of TUR-BT specimens according to clinical diagnostic modalities.

	Overall (n = 245)		No cancer (n = 42)		Histologic grade				
					Low-grade (n = 36)		High-grade (n = 143)		*p* value
	n	%	n	%	n	%	n	%	
**MRI T staging**									
No lesion	52	21	20	48	5	14	14	10	< 0.001
≤ T1	125	51	16	38	26	72	73	51	
≥ T2	68	28	6	14	5	14	56	39	
**Urine cytology**									
Unsatisfactory	10	4	1	2	2	6	7	5	< 0.001
Negative for HGUC	86	35	30	71	27	75	24	17	
Atypical urothelial cell	44	18	4	10	4	11	31	22	
Suspicious for HGUC	31	13	1	2	2	6	22	15	
HGUC	74	30	6	14	1	3	59	41	

*MRI* magnetic resonance imaging, *TUR-BT* transurethral resection of bladder tumor.

### Performance of noninvasive diagnostic modalities

Sensitivity, specificity, PPV, NPV, accuracy, DOR, and NNM are described in Table 4 and Fig. 1. The combined MRI and cytology group showed the highest sensitivity, NPV, accuracy, DOR, and NNM, whereas the cytology-alone group revealed the highest specificity and PPV. There was no significant difference in the performance of noninvasive diagnostic modality according to the number of previous surgeries and intravesical therapies (Table 5a). However, diagnostic performance for the group of patients who has undergone MMC more than 1 year before was the lowest (Table 5b).

**Table 4:** Performance of noninvasive diagnostic modalities to diagnose bladder cancer based on the pathology of TUR-BT specimens.

	MRI (n = 245)	Cytology (n = 245)	MRI + Cytology (n = 245)
Sensitivity	84% (81–87)	68% (65–70)	96% (93–98)
Specificity	48% (34–61)	74% (59–85)	43% (31–53)
PPV	89% (86–92)	93% (89–96)	89% (87–91)
NPV	38% (27–49)	32% (26–37)	67% (48–82)
Accuracy	78% (73–83)	69% (64–73)	87% (82–90)
DOR	4.9 (2.2–11)	6.0 (2.7–14)	16 (6.0–44)
NNM	4.5 (3.7–5.7)	3.2 (2.8–3.7)	7.4 (5.7–9.8)

*PPV* positive predictive value, *NPV* negative predictive value, *DOR* diagnostic odds ratio, *NNM* number needed to misdiagnose, *MRI* magnetic resonance imaging, *TUR-BT* transurethral resection of bladder tumor.

**Table 5:** Performance of noninvasive diagnostic modalities.

	Times of operation ≤ 3 (n = 196)	Times of operation > 4 (n = 49)	No intravesical therapy (n = 55)	Previous MMC (n = 48)	Previous BCG (n = 142)
**(a) Performance according to the number of previous surgeries and intravesical therapies**					
Sensitivity	96% (94–98)	92% (84–98)	93% (82–99)	89% (75–97)	98% (94–100)
Specificity	39% (25–49)	55% (28–73)	44% (14–79)	45% (17–77)	41% (21–64)
PPV	89% (87–91)	88% (80–93)	89% (82–94)	89% (82–93)	89% (85–92)
NPV	67% (43–85)	67% (34–90)	58% (27–84)	46% (22–73)	83% (54–96)
Accuracy	87% (83–91)	84% (72–92)	85% (73–93)	82% (68–91)	89% (82 = 93)
DOR	17 (5.1–57)	14 (2.1–109)	11 (1.5–98)	6.9 (1.1–45)	41 (7.0–310)
NNM	7.8 (5.9–11)	6.1 (3.5–13)	6.9 (4.3–14)	4.8 (3.1–9.2)	9.5 (6.5–12)

	Previous MMC ≤ 1 yr (n = 22)	Previous MMC > 1 yr (n = 26)	Previous BCG ≤ 1 yr (n = 59)	Previous BCG > 1 yr (n = 83)	
**(b) Performance according to type and term between intravesical treatement and noninvasive diagnostic modalities**					
Sensitivity	100% (79–100)	81% (58–95)	98% (90–100)	99% (92–100)	
Specificity	67% (22–96)	20% (1–72)	40% (12–74)	42% (15–72)	
PPV	94% (82–98)	83% (75–89)	89% (83–93)	89% (84–93)	
NPV	100 (not applicable)	18% (3–61)	80% (33–97)	86% (44–98)	
Accuracy	94% (76–100)	71% (50–87)	88% (77–95)	89% (80–95)	
DOR	Not applicable	1.1 (0–17)	41 (7–310)	50 (4.4–1315)	
NNM	11 (3–11)	3.3 (2.6–7.0)	9.5 (6.5–12)	10 (6.2–14)	

## Discussion

Through this study, we explored the potential of replacing cystoscopy with mpMRI and cytology in NMIBC follow-up. These tests still present some limitations for clinical use; however, in the future it can be considered as noninvasive test when decreased costs and technological advances make their application more feasible.

Cystoscopy currently remains the gold standard for the follow-up of patients with bladder cancer^12^. At the first year of diagnosis for bladder cancer, patient has to undergo cystoscopy for every 3–4 months and 6 months to annually thereafter. However, side effects remain even after several efforts for improvement, especially for patient anxiety^13–17^. Additionally, cystoscopy is a time-consuming and burden-presenting procedure for practitioners and caregivers.

Bladder MRI has the advantage of reproducibility of the image over time and can obtain objective information about the size, depth, and number of tumors. Simultaneously obtaining information about the upper urinary tract and surrounding organs such as the prostate, uterus, or ovary may result in better treatment^18^.

**Figure 6:**
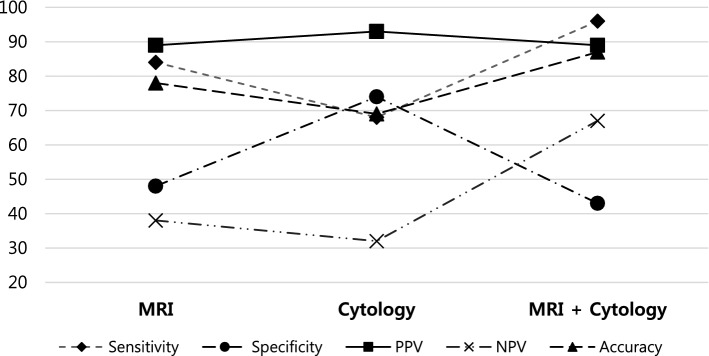
Sensitivity, specificity, positive predictive value (PPV), negative predictive value (NPP), and accuracy of diagnostic modalities for pathologically confirmed bladder cancer.

In our study, for pathologically confirmed bladder cancer lesions, the sensitivity, NPV, and accuracy of diagnosis using MRI combined with cytology were higher than when using MRI alone (Table 4). This also exceeded the sensitivity of 57–87% reported in a prospective study with white light cystoscopy and was similar to the sensitivity of 93–100% observed using narrow-band image cystoscopy^19,20^. When compared with the diagnostic method, it has been confirmed that when MRI and cytology results are combined, the sensitivity, NPV, and accuracy increased significantly, whereas there was no significant difference in PPV. However, specificity was shown to be at the lowest level for the combined diagnostic method which maybe reason for overtreatment (Fig. 6). Even so, since all the subjects included in this study were patients who had decision for additional surgery based on cystoscopic findings, the issue for overtreatment rate between cystoscopy and non-invasive study may require additional comparison in the future. Our study has focused on not missing abnormal lesions with combined MRI and cytology. Therefore, sensitivity was thought to be more valuable than specificity, which has been achieved and proven by the performance of the combined diagnostic methods of MRI and cytology.

Despite the abovementioned advantages, bladder MRI has several limitations. Most important of all, because MRI is a time-consuming and expensive test, it is difficult to apply to all patients and institutions. It is difficult to perform in patients with claustrophobia or with a pacemaker; an accurate examination is difficult for collapsed bladder, and adverse reactions may occur with the use of contrast agents. Additionally, it may be difficult to recognize flat lesions as CIS. In our study, 24 cases were diagnosed only as pTis without synchronous pT1–4 lesions. Diagnosis by MRI alone showed a sensitivity of 46% and NPV of 61%, while diagnosis by combined MRI and cytology showed a sensitivity of 92% and NPV of 90% (Table 6). As a diagnostic tool for pTis, the results obtained by using combined MRI and cytology may be better than findings from previous studies using white light and narrow-band imaging^19,20^. However, as previously mentioned about MRI, cytology is also a test that requires a lot of time, cost and manpower. Therefore, it seems hard to say that combined MRI and cytology are better tests than cystoscopy in terms of cost and efficiency.

**Table 6:** Performance of noninvasive diagnostic modalities for pathologically confirmed Tis lesion without synchronous pT1-4 lesions.

	MRI (n = 66)	Cytology (n = 66)	MRI + Cytology (n = 66)
Sensitivity	46% (29–63)	79% (62–91)	92% (75–99)
Specificity	48% (38–58)	74% (64–81)	43% (34–47)
PPV	33% (21–46)	63% (49–73)	48% (39–51)
NPV	61% (48–73)	86% (74–94)	90% (71–98)
Accuracy	47% (35–60)	76% (63–85)	61% (49–66)
DOR	0.77 (0.25–2.4)	11 (2.8–43)	8.3 (1.5–58)
NNM	1.9 (1.5–2.5)	4.1 (2.7–6.5)	2.5 (2.0–2.9)

According to the NCCN guideline, although it depends on the risk group, an annual upper tract imaging follow-up may be required after primary TUR-BT, which can be done simultaneously while undergoing bladder MRI. In addition, even it is a rare case, extra-vesical growth of bladder cancer which cannot be detected by cystoscopy may be detected by MRI. Although follow up by MRI may be difficult for regular follow-up in real world, when upper tract imaging is required during follow-up or when the patient wants noninvasive follow up test despite of the cost or for other various reasons, MRI can be considered as an alternative follow up test for bladder cancer for patients.

Another important issue is that repeated TUR-BT and mucosal irritating intravesical therapy may lead to misinterpretation of bladder MRI findings. Comparing groups by the history of the number of TUR-BT sessions and MMC or BCG instillation, there were no significant differences in the diagnostic performance (Table 5). However, our study population had only 15% of patients who had undergone intravesical therapy within 6 months prior to MRI imaging. Therefore, the possibility of misinterpretation may have been muted.

In our study, among the nine cases that were not detected by MRI or cytology, three (1.2%) were falsely interpreted by the radiologist and two (0.8%) had an empty bladder during MRI. Three patients (1.2%) had symptoms that required cystoscopy. Only one case (0.4%) could not have been diagnosed as a cancerous lesion. However, this patient was found to have an extremely small low-grade inverted variant; active surveillance could have been considered for this case.

As can be seen from our results, the possibility for misdiagnosis of cancer recurrence by using only non-invasive tests still exists, even if it is extremely low. However, the probability of misdiagnosis using cystoscopy appears to be similar. Bladder MRI was performed as an additional test for patients diagnosed by cystoscopy in our study; hence, it is difficult to determine the number of cases diagnosed solely by MRI. However, previous studies found that the active surveillance of small lesions may be a safe management strategy, because the risk of progression to recurrence and muscle invasion is low in patients with NMIBC, which gives us a safety margin for lesions that are difficult to detect by several methods^21–24^.

Because of high-cost and low-availability of MRI, high-resolution micro-ultrasound (mUS), which has recently been used in some institutions. Therefore, with the mUS, various attempts and studies are being made in the fields of prostate and bladder cancer. Pietro et al., reported that, in a retrospective study of primary diagnosis of BC, sensitivity of mUS was higher for detecting muscle invasion of bladder cancer than bladder MRI. Of course, there are disadvantages that the accuracy of the examination differs depending on how experienced the physician is and the patient's obesity level or the shape of the prostate in the bladder^25^.

To the best of our knowledge, this is the first study to evaluate the efficacy of using only noninvasive tests for the follow-up of patients with bladder cancer. However, there are several limitations of this study. First, NMP22 biomarker analysis was not included in our study because of the lack of data. Second, cytology was performed during cystoscopy, which may have led to a higher diagnostic rate because it corresponds to bladder washing cytology.

## Conclusion

In our experience, for the follow-up of NMIBC, the sensitivity of combined noninvasive tests (bladder MRI and urine cytology) was comparable to cystoscopy for diagnosing recurrent lesions but not for specificity. However, it may reduce the need for cystoscopy and allowing patients to have choices for follow up diagnostic methods. Also, additional imaging tests to evaluate kidney, ureter and peri-vesical lesions can be reduced. Further studies aiming to reveal the diagnostic performance of noninvasive studies in a larger population are mandatory before its introduction in clinical practice.

## Data Availability

The anonymized data is available upon reasonable request towards the corresponding author.
